# White Matter Hyperintensity Regression: Comparison of Brain Atrophy and Cognitive Profiles with Progression and Stable Groups

**DOI:** 10.3390/brainsci9070170

**Published:** 2019-07-19

**Authors:** Omar M. Al-Janabi, Christopher E. Bauer, Larry B. Goldstein, Richard R. Murphy, Ahmed A. Bahrani, Charles D. Smith, Donna M. Wilcock, Brian T. Gold, Gregory A. Jicha

**Affiliations:** 1 Sanders-Brown Center on Aging, University of Kentucky, Lexington, KY 40536, USA; 2 Department of Behavioral Science, College of Medicine, University of Kentucky, Lexington, KY 40508, USA; 3 Department of Neurology, College of Medicine, University of Kentucky, Lexington, KY 40508, USA; 4 Department of Neuroscience, College of Medicine, University of Kentucky, Lexington, KY 40508, USA; 5 Department of Biomedical Engineering, College of Engineering, University of Kentucky, Lexington, KY 40506, USA; 6 Department of Physiology, Colleges of Medicine, University of Kentucky, Lexington, KY 40536, USA

**Keywords:** white matter hyperintensities, WMH regression, WMH progression, Stable WMH, ADNI, brain atrophy, cognition

## Abstract

Subcortical white matter hyperintensities (WMHs) in the aging population frequently represent vascular injury that may lead to cognitive impairment. WMH progression is well described, but the factors underlying WMH regression remain poorly understood. A sample of 351 participants from the Alzheimer’s Disease Neuroimaging Initiative 2 (ADNI2) was explored who had WMH volumetric quantification, structural brain measures, and cognitive measures (memory and executive function) at baseline and after approximately 2 years. Selected participants were categorized into three groups based on WMH change over time, including those that demonstrated regression (*n* = 96; 25.5%), stability (*n* = 72; 19.1%), and progression (*n* = 209; 55.4%). There were no significant differences in age, education, sex, or cognitive status between groups. Analysis of variance demonstrated significant differences in atrophy between the progression and both regression (*p* = 0.004) and stable groups (*p* = 0.012). Memory assessments improved over time in the regression and stable groups but declined in the progression group (*p* = 0.003; *p* = 0.018). WMH regression is associated with decreased brain atrophy and improvement in memory performance over two years compared to those with WMH progression, in whom memory and brain atrophy worsened. These data suggest that WMHs are dynamic and associated with changes in atrophy and cognition.

## 1. Introduction

Magnetic resonance imaging (MRI) cerebral white matter hyperintensities (WMH) are non-specific, subcortical signal regions with prolonged T2 relaxation times compared to normal appearing white matter. They often appear conspicuously bright on T2-weighted images, particularly fluid-attenuated inversion recovery (FLAIR) images where nearby bright cerebrospinal fluid intensity is suppressed. Late-life WMH are thought to largely represent cerebrovascular injury resulting from cerebral small vessel disease (cSVD) [1]. Such injury may lead to neuronal circuit dysfunction in affected areas that can be associated with vascular cognitive impairment and dementia. Several previous studies demonstrate an association between progression of WMH lesion volume and worsening cognitive impairment over time [2,3,4,5]. Although some studies have reported cases of WMH volume decrease over time (regression), the relationship of these changes with cognitive outcomes needs to be further evaluated [6,7]. To date, the only study that examined the relationship between the WMH regression and structural brain changes and cognitive function is from the RUN DMC cohort [8]. The latter study concluded that WMH regression group showed similar cognitive decline when compared to the WMH stable group. In addition, WMH progression group showed more cognitive decline when compared to the WMH stable group. Both WMH regression and stable groups showed no brain atrophy, suggesting a relatively benign prognosis of such groups.

It is possible that WMH regression reflects imaging or methodological confounders rather than a true biological phenomenon [9]. The use of standardized imaging sequences, scanners, and head coils across longitudinal visits, in addition to regular scanner calibration and identical processing techniques with uniform intensity corrections, such as that used in the Alzheimer’s Disease Neuroimaging Initiative (ADNI) study, are required to exclude analytic confounders and imaging artifacts that could be interpreted as representing WMH regression. ADNI was launched in 2003 as a public/private partnership designed to assess biological and clinical markers of Alzheimer’s Disease (AD) progression (http://adni.loni.usc.edu).

Biologically, it is possible that regression of WMH volume represents gliotic contraction and/or microvascular encephalomalacia resulting from static ischemic injury. If WMH regression represents such a biological phenomenon, it could be associated with greater global brain atrophy and unchanged or diminished cognitive function as occurs in many individuals post-stroke. Alternatively, WMH regression might reflect a longitudinal reduction in inflammatory changes and focal edema associated with cSVD, and if so, such changes should be associated with improved cognition despite a reduction in total brain volume. Lastly, it is possible that WMH regression represents healing or regenerative processes, that would be associated with reduced brain atrophy and improvement in cognitive performance. A theoretical framework to summarize the course and interpretation of such longitudinal changes are provided in Table 1.

The present study explored hypothetical mechanisms underlying WMH regression by examining cognitive and structural brain changes (i.e., brain atrophy) that occur in the setting of WMH volume regression through the analysis of longitudinal data collected as part of the ADNI 2 study.

## 2. Materials and Methods

### 2.1. Study Population

The study cohort was comprised of 351 ADNI 2 participants with normal cognition or mild cognitive impairment who had WMH quantification both at baseline and at 2-years +/− 3 months [10]. Inclusion criteria included the availability of complete demographic information, diagnostic information within 1 year of the T2 FLAIR scan used for WMH quantification at baseline, a T1-weighted (MPRAGE) image and FreeSurfer structural segmentation [11,12] within 1 year of the baseline FLAIR images, atrophy composite scores within 1 year of the baseline FLAIR images, and neurocognitive composite metrics for assessments of both memory [13] and executive function (EF) [14]. Potential ADNI participants not meeting these criteria were excluded. The memory composite included the Rey auditory verbal learning test (RAVLT), the cognitive component of the Alzheimer’s disease assessment scale (ADAS-Cog), the Folstein mini-mental state examination (MMSE), and Wechsler logical memory scale scores, whereas the executive function composite included the clock drawing test, trail making test, category fluency (animal and vegetable), Wechsler adult intelligence scale-revised (WAIS-R) digit span and digit symbol tests. Details of ADNI clinical procedures and methodology are available elsewhere [15,16].

### 2.2. MRI Acquisition

FLAIR images were acquired according to standard ADNI protocols [15]. Scanning parameters were identical within participants between the two-time points. As a result, the reported WMH progression, stability, and regression measures fulfill the criteria needed to control for radiological and methodological confounders [17]. Further information can be found at http://adni.loni.usc.edu.

### 2.3. White Matter Hyperintensity Calculations

WMH volumes were calculated using the 4-tissue segmentation method [18]. Briefly, FLAIR images were co-registered to the T1 image, inhomogeneity-corrected and non-linearly aligned to a minimal deformation template (MDT) using the T1 transformation and the FMRIB Software Library (FSL) toolbox [19,20]. WMHs were estimated in MDT space using Bayesian probability and prior probability maps [18]. Binary WMH masks were created using a threshold of 3.5 SD above the mean. Volume of WMH were then calculated after back-transformation into native space. Gray, white, and CSF measurements were segmented using an expectation–maximization algorithm. WMH were ultimately subtracted from segmented white matter volume and reported in cubic millimeters.

### 2.4. Longitudinal Change Calculations

Changes in the various measures (Δ) were calculated by subtracting the baseline value from the value at the 2-year follow-up. Positive values indicate increases whereas negative values indicate decreases between the two time points. For WMH volume change, a negative value greater than 150 mm^3^ indicates regression (i.e., less WMH volume at follow-up) whereas a positive value greater than 150 mm^3^ indicates progression (i.e., more WMH volume at follow-up). Ten participants did not have follow-up neurocognitive measures and 1 did not have ventricular volume within the 2 years +/− 3-month range; these values were excluded from further analyses.

### 2.5. WMH Categorization

**Figure 1:**
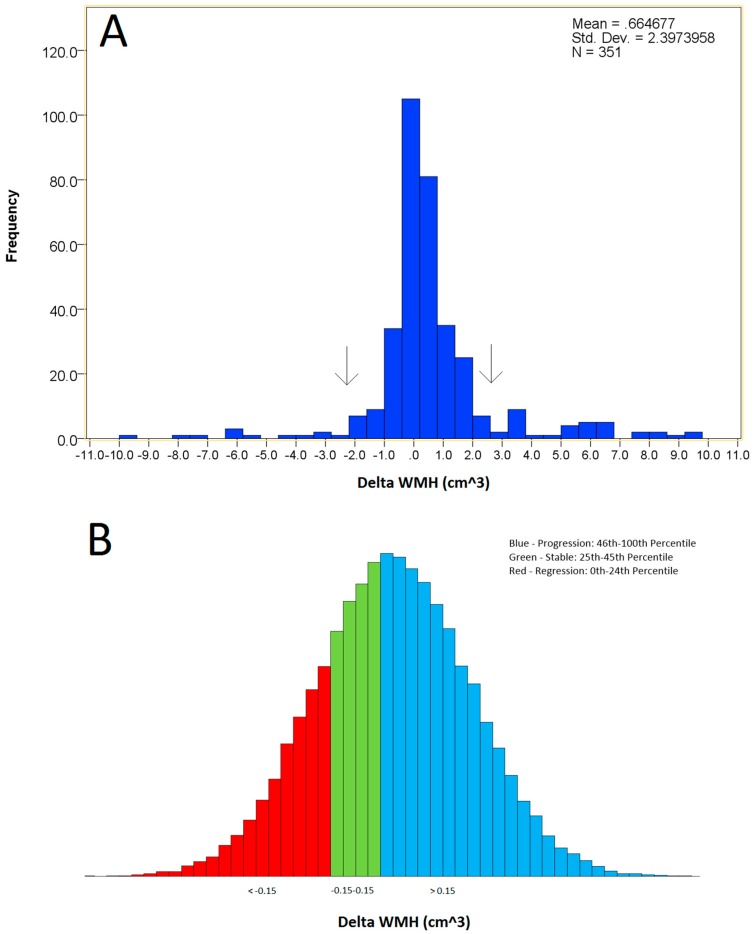
White matter hyperintensity distribution in the sample studied. (**A)** The true distribution of the data, showing notable leptokurtosis. Black arrows indicate standard deviation, demonstrating why standard deviation was not deemed an appropriate criterion for separating groups. (**B**) Divisions of the three white matter hyperintensity (WMH) groups. Visualization only.

WMH net volume change between the baseline and the follow-up visits was used to calculate Δ WMH. Participants were initially grouped based on Δ WMH volume (regression, stable, and progression) using a percentile-based approach in which the percentile for no change was first identified (the 35th percentile). Although definitions based on standard deviations were initially considered, the notable leptokurtic distribution (Figure 1) of the data precluded such classification as only the most extreme values would be defined outside of the stable group. Ultimately, we defined the stable group as representing the +/− 10th percentile from the percentile of no change (25th–45th percentile of the study participant distribution). This corresponded to +/− Δ of 150 mm^3^ of WMH lesion volume. Participants classified in the regression group had reductions in WMH volume greater than 150 mm^3^ (more than 10 percentiles below the percentile of no change) and those classified in the progression group had an increase in WMH volume greater than 150 mm^3^ (more than 10 percentiles above the percentile of no change).

### 2.6. Atrophy Composite Calculation

To quantitate changes in brain and ventricular volume to estimate changes in global brain atrophy that may be related to WMH changes, gray and white matter segmentation volumes [18] from the four-tissue ADNI classification were combined to produce a total brain volume (cm^3^). However, this global measure alone does not specifically account for the volumetric changes in the ventricles, which is associated with both WMH changes [21] and AD-related neurodegenerative processes [22]. Therefore, the volume of the lateral ventricles (cm^3^) was estimated using FreeSurfer (University of California at San-Francisco, San-Francisco, CA, USA). Although these measures were assessed individually, the final measure was a composite that used the z-scores of brain volume subtracted from the z-scores from the lateral ventricles (higher value means less brain volume and/or larger ventricles). This was done as an attempt to account for both periventricular and subcortical atrophy (ventricular volume) as well as more cortical-based whole brain atrophy (brain volume).

### 2.7. Statistical Analysis

All statistical analyses were conducted using SPSS (version 24, SPSS Inc., Chicago, IL, USA). All dependent variables were approximately normally distributed and screened for outlier removal. Outliers were removed if they were 3 standard deviations outside the mean, specifically, three data points in Δ Memory, three in Δ EF, seven in Δ Ventricular Volume, and three in Δ Brain Volume; any outliers removed from the latter two were not entered into Δ Atrophy.

ANOVAs were used to compare age and education between WMH progression, regression, and stable groups. Pearson’s Chi Square was used to examine differences between groups in sex, marital status, and cognitive status (normal, mild cognitive impairment [MCI], and AD). Between-group differences in Δ atrophy composite, Δ ventricular volume, Δ brain volume, Δ memory, and Δ EF between the 3 groups were examined using ANCOVA with both age and sex as covariates. All statistics were considered significant at *p* < 0.05 and were false discovery rate (FDR) corrected using the Benjamini–Hochberg procedure [23].

## 3. Results

Participants were 72 ± 7.2 years old (48.3% women), with 16.5 ± 2.6 years of education. Clinical cognitive status was normal in 40.2% and MCI in 59.8% (Table 2). There were no significant differences between regression, stable, and progression groups in age, education, sex, marital status, diagnosis, or Δ EF. Participants were classified as having WMH regression in 25.5%, no change in 19.1%, and WMH progression in 55.4%.

ANCOVA revealed that there were differences in Δ atrophy composite between groups (*p* = 0.005), particularly the progression and regression (*p* = 0.041) and the progression and stable groups (*p* = 0.003, Table 2). Longitudinally, memory improved in the regression and stable groups compared to progression (*p* = 0.036; *p* = 0.036 respectively, Table 3). There were no differences between any groups in Δ EF (*p* = 0.492, Table 3).

## 4. Discussion

**Figure 2:**
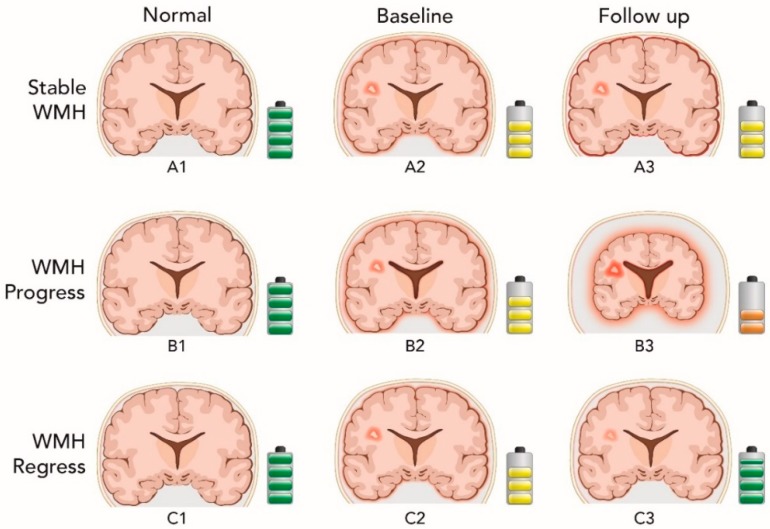
Descriptive figure showing the dynamic nature of the white matter hyperintensity changes over time and the associated change in brain volume and cognitive performance. The coronal image cartoon represents the brain and ventricle size (scale of change exaggerated for emphasis), while the battery represents memory performance (improved memory corresponding to more battery capacity and green). The first column represents a normal brain with no WMH and normal memory, the second and third columns the baseline and two-year follow up showing changes over the course of the study. The upper row represents WMH stability. Brain volume and memory remained stable in the follow up visit as shown in A2 and A3. The middle row illustrates the case of progression of WMH over time. In B3, the WMH region enlarges, brain volume decreases along with ventricular increases and memory declines compared to B2. Finally, the lower row represents cases of WMH regression over time. In C3, the WMH lesion volume shrinks, brain volume is maintained, and memory is improved when compared to C2.

These data indicate that WMH regression is associated with decreased brain atrophy and cognitive decline over a period of two years compared to WMH progression, which, consistent with prior reports, is associated with increased brain atrophy [24] and cognitive decline [25,26,27] (Table 2 and Figure 2).

WMH progression and regression have been reported in smaller cohorts [6,7,22,28], and a stable group has also been identified in other studies [7,8,22]. Several studies reported that WMH progression is associated with greater cognitive decline [4,5,8]; however, only one study reported on the potential associations between WMH regression and changes in global atrophy or cognitive test performance over time. The latter study showed that WMH regression was not associated with brain atrophy and was associated with cognitive decline similar to that seen in the WMH stable group, suggesting a relatively benign cognitive outcome [8].

Our data shows that, although there were significant differences in atrophy and cognition between those with WMH progression and regression, and between those with progressive and stable WMHs, there were no differences between those with WMH regression and stability. Both WMH progression and regression groups had larger baseline WMH volume than the stable group. This implies that at least some of those with WMH injury can return toward a normal functioning state.

We used the conventional net change in the WMH between the baseline and the follow-up visits together with the percentile approach to categorize the dynamic changes into three groups (progression, stable, and regression). We found that about 25.5% of study participants showed WMH regression, which is similar to the 11.3% and 21.5% reported by others [8,29]. In addition, 19.1% of the cohort remained stable and 55.4% showed WMH progression over two years. These data add to the existing literature suggesting that WMH volume change is a dynamic process.

We had several possible hypotheses regarding possible explanations for WMH regression. First, it could be due to imaging acquisition or methodological confounders such as the use of different scanners across visits, lack of scanner calibration, lack of standardized acquisition parameters, differences in post-acquisition processing techniques such as registration or segmentation pipelines, all of which may affect the accuracy of the WMH volume change calculation [9]. Use of the ADNI cohort and WMH volumes from the validated UCD four-tissue segmentation method for this study minimized such effects, suggesting instead that the observed dynamic nature of longitudinal WMH volume changes reflects a real biological phenomenon.

**Figure 3:**
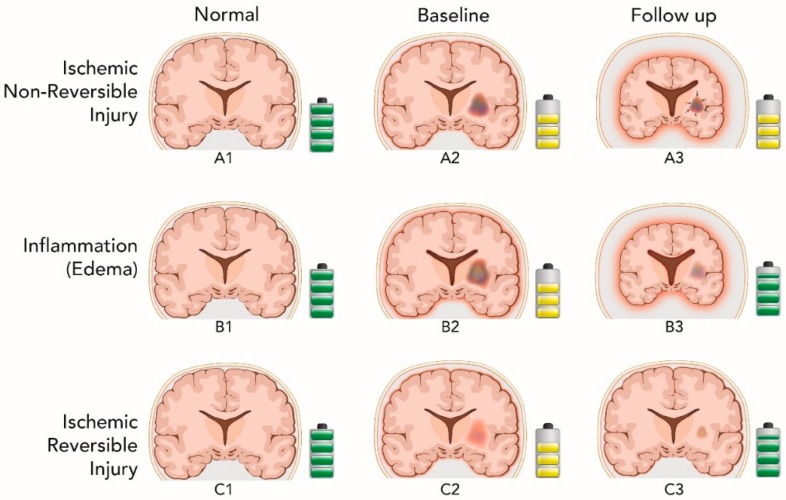
Conceptual descriptive figure showing the possible mechanisms that lead to white matter hyperintensity regression overtime and their associated effect on the brain volume and the cognitive performance (same conventions as in Figure 2). The upper row demonstrates the first potential mechanism of white matter hyperintensity (WMH) regression due to ischemic non-reversible injury. In A3, the WMH lesion contracts (black arrows) resulting in brain volume reduction compared to A2. Because the contraction is merely mechanical, memory performance is not changed compared to A2. The middle row illustrates inflammation as a potential cause of WMH regression. In B3, the edema resolves, the brain volume consequently decreases but memory is improved compared to B2. The difference between A3 and B3 is in expected memory performance. Lower row represents a third explanation of WMH regression, most consistent with our data. In C3, WMH lesion volume regresses, but brain volume and memory performance increase compared to C2. The difference between C3 and B3 is in brain atrophy, which is greater in B3.

Biological causes for WMH regression could include gliotic scarring and microstructural encephalomalacia due to irreversible ischemic parenchymal injury, resolution of secondary inflammatory processes as a result of irreversible ischemic damage, or could instead reflect resolution or healing of reversible ischemic injury. The associations of WMH regression with measures of global cerebral atrophy and cognitive change over time would be expected to differ between these possibilities as highlighted in Table 1 and Figure 3.

If WMH regression were the result of gliotic scarring and microstructural encephalomalacia, such change could be associated with increased cerebral atrophy and stable or worsening cognition. In such a scenario, acute small subcortical strokes and lacunar infarcts, which mimic WMH, could account for WMH regression through the natural course of temporal evolution. Such permanent lesions eventually reduce in diameter over time, which could in part account for the observed WMH regression seen in this and other studies [22,30]. The current data, however, argue against this possibility as retraction and temporal evolution of ischemic lesions would be expected to result in increased rather than the decreased global brain atrophy observed in our study.

It is also possible that WMH regression may be due to resolution of inflammatory processes and focal edema related to irreversible ischemic injury. This hypothesis is supported by prior work demonstrating that parenchymal edema develops early during the WM vascular injury process [31]. Several studies supporting the concept of resolving edema as an explanation for WMH regression are based on observations in patients with cerebral autosomal dominant arteriopathy with subcortical infarcts and leukoencephalopathy (CADASIL) or stroke [32]. If this possibility were responsible for WMH regression, measures of atrophy would increase in the setting of WMH regression as inflammatory edema resolved, and cognitive function would likely stabilize or improve. Although this is a possibility for some WMH lesions, the overall lack of increased atrophy argues against this mechanism as a primary determinant of WMH regression.

Lastly, it is possible that WMH regression is most closely related to resolution of reversible ischemic changes or to healing or regenerative processes after such injury. In such a scenario, WMH regression would be associated with either no change or increased brain volume, and would also be associated with improvement in cognitive test performance. This hypothesis is best supported by our observations.

Our data highlight the importance of considering WMH change over time as a dynamic process, which can be related to both positive and negative structural and cognitive outcomes. Future work should explore the many demographic, risk, and treatment variables that may have influenced longitudinal WMH change in our cohort of subjects, perhaps suggesting disease modifying strategies to prevent or reverse vascular cognitive impairment and dementia. Further longitudinal studies are also needed to determine if there is interchange between groups—alternation between WMH progression and regression states over time for example.

Limitations of the study involve time of observation in a chronic process, generalizability, and sensitivity of measures. The ADNI cohort has unique characteristics based on its inclusion/exclusion criteria, which include an emphasis on earlier stage disease including normal and MCI subjects as well as criteria that excludes those with significant cerebrovascular disease and/or CVD risk factors. As such, the cohort is not generalizable to epidemiologic and community-based cohorts that may have higher proportions of cognitively impaired individuals and/or those with increased cerebrovascular risk factors and/or WMH burden. We used global net change in the WMH between the baseline and the follow up visits, limiting our ability to detect regional change in WMH progression, stability, or regression compared to more detailed spatial location analyses used by others previously [22]. Finally, we only used two time points for demonstrating the dynamic nature of WMH; more sampling of the time evolution curve of WMH is desirable in future.

Despite these limitations, the present study allowed an exploration of cognitive and brain volume changes that are associated with dynamic WMH changes. The present data demonstrate that WMH regression is not a mere imaging artifact or artifact due to methodologic procedures, but rather represents a real biological phenomenon that may, at least in part, reflect recovery from and resolution of reversible cerebrovascular injury.

## 5. Conclusions

Our data indicate that WMH regression is present in at least a quarter of this ADNI sample. Those with WMH regression had a reduction in cognitive decline and decreased brain atrophy compared to those with WMH progression, with no differences in either atrophy or memory performance between the regression and stable groups. Identifying the underlying mechanisms that result in WMH regression, and how these mechanisms may be promoted to produce more favorable clinical outcomes should be further investigated.

## Figures and Tables

**Table 1:** Possible etiologies for cerebrovascular-related white matter hyperintensities that regress over time and the expected associations with cerebral atrophy and cognitive performance.

Possible Etiology for WMH	Potential Cause of Regression	Expected Association with Cerebral Atrophy	Expected Association with Cognitive Performance
Irreversible ischemic injury	Gliotic contraction and microscopic encephalomalacia	Increased atrophy	No change in cognitive performance
Inflammation associated with irreversible ischemic injury	Resolution of inflammation and edema with restoration of normal function in the penumbra	Increased atrophy secondary to reduced inflammatory edema	Improvement in cognitive performance
Reversible ischemic injury	Healing process	Decreased atrophy	Improvement in cognitive performance

**Table 2:** Demographic, clinical, imaging, and change scores for subjects demonstrating progression, stability, and regression in white matter hyperintensity volumes. WMH is reported in cubic centimeters; Memory and executive function (EF) are reported in standardized scores, and Atrophy composite, brain volume, and ventricular volume are z-scored. Raw scores for brain and ventricular volume per group can be viewed in Table S1.

Criteria	Progressors (*n* = 190)	Regressors (*n* = 93)	Stable (*n* = 68)	Significance	*n*; (Progressors, Regressors, Stable)
Age; (mean, SD)	72.2 (6.9)	72.0 (7.3)	70.3 (7.2)	0.163	190, 93, 68
Education; (mean, SD)	16.4 (2.6)	16.7 (1.6)	16.7 (2.4)	0.654	190, 93, 68
Female; (*n*, %)	98 (51.6)	40 (43.0)	34 (50.0)	0.393	190, 93, 68
Currently Married; (*n*, %)	140 (73.7)	72 (77.4)	50 (73.5)	0.764	190, 93, 68
Cognitively Normal; (n, %)	71 (37.4)	38 (40.9)	32 (47.1)	0.371	190, 93, 68
MCI (*n*, %)	119 (62.6)	55 (59.1)	36 (52.9)	0.371	190, 93, 68
Baseline WMH; (mean, SD)	6.9 (10.3)	8.2 (10.6)	1.9 (2.0)	-	190, 93, 68
Follow Up WMH; (mean, SD)	8.7 (11.6)	6.9 (9.5)	1.9 (2.0)	-	190, 93, 68
Δ Memory; (mean, SD)	−0.07 (0.35)	0.02 (0.32)	0.05 (0.32)	-	184, 89, 65
Δ EF; (mean, SD)	−0.06 (0.59)	0.00 (0.62)	0.04 (0.56)	-	182, 90, 65
Δ Atrophy Comp; (mean, SD)	0.19 (1.40)	−0.17 (1.36)	−0.52 (1.08)	-	179, 90, 63
Δ Brain Volume; (mean, SD)	−0.07 (0.90)	0.15 (1.0)	0.18 (0.78)	-	187, 93, 68
Δ Ventricular Volume; (mean, SD)	0.12 (0.94)	−0.08 (0.69)	−0.34 (0.65)	-	182, 90, 63

Abbreviations: *n*, number; SD, standard deviation; MCI, mild cognitive impairment; AD, Alzheimer’s disease; WMH, white matter hyperintensities; EF, executive function composite.

**Table 3:** ANCOVA results examining brain volume composite, memory change, and EF change in all three groups. Age and gender were used as covariates.

ANCOVA	Post-Hoc Comparisons	*p*-Value	*p*-Value (FDR-Corrected)
Dependent Variable			
Δ Memory		0.017 *	0.028 *
	Progression/Regression	0.024 *	0.036 *
	Progression/Stable	0.019 *	0.036 *
	Regression/Stable	0.766	0.766
Δ EF		0.492	0.492
	Progression/Regression	0.398	0.398
	Progression/Stable	0.293	0.293
	Regression/Stable	0.790	0.790
Δ Atrophy Composite		0.001 ^‡^	0.005 **
	Progression/Regression	0.027 *	0.041 *
	Progression/Stable	0.001‡	0.003 **
	Regression/Stable	0.172	0.172
Δ Ventricular Volume		0.011 *	0.028 *
	Progression/Regression	0.036 *	0.054
	Progression/Stable	0.007 **	0.021 *
	Regression/Stable	0.443	0.433
Δ Brain Volume		0.061	0.076
	Progression/Regression	0.054	0.090
	Progression/Stable	0.060	0.090
	Regression/Stable	0.887	0.887

* indicates significant at *p* < 0.05. ** indicates significant at *p* ≤ 0.01. ^‡^ indicates significant at *p* ≤ 0.001. Abbreviations: WMH, white matter hyperintensities; EF, executive function composite.

## Supplementary Materials

The following are available online at https://www.mdpi.com/2076-3425/9/7/170/s1, Table S1: Raw measurements (cubic centimeters) in the three groups for brain and ventricular volume.
