# Supplementary material for: White Matter Hyperintensity Regression: Comparison of Brain Atrophy and Cognitive Profiles with Progression and Stable Groups
Source: Brain Sci. 2019 Jul 19;9(7):170. doi: 10.3390/brainsci9070170 (PMC6680735; doi:10.3390/brainsci9070170)
Supplement: Supplementary file 1 [file brainsci-09-00170-s001.pdf]

Table S1. Raw measurements (cubic centimeters) in the three groups for brain and ventricular volume.

Criteria	Progressors (n = 190)	Regressors (n = 93)	Stable (n = 68)
**Δ Brain Volume (mean, SD)**	−5.4688 (19.0)	−0.7783 (16.6)	−0.1384 (22.1)
**Δ Ventricular Volume (mean, SD)**	4.031 (3.5)	3.279 (2.6)	2.321 (2.436)
